# Vaginal Angiolipoleiomyoma: A Case Report and Literature Review

**DOI:** 10.7759/cureus.107341

**Published:** 2026-04-19

**Authors:** Diana Mendoza-Arcique, Guillermo Del Rio-Bojorquez, Fernando Interián-Alvarez, Brayan J Ortiz-Villanueva, Jose M Erosa-Gonzalez, Jocelyn Pumares-Campos, Darinela Y Borges-Marquez, Cesar Herrera-Mendez

**Affiliations:** 1 Department of Gynecology and Obstetrics, Hospital Regional "Elvia Carrillo Puerto" Institute for Social Security and Services for State Workers (ISSSTE), Mérida, MEX; 2 Faculty of Medicine, Universidad Autónoma de Yucatán, Mérida, MEX; 3 Mexican Faculty of Medicine, La Salle University, Mexico City, MEX; 4 Department of Gynecological Endoscopic Surgery, Hospital Regional Adolfo López Mateos, Institute for Social Security and Services for State Workers (ISSSTE), Mexico City, MEX

**Keywords:** angiolipoleiomyoma, case reports, immunohistochemistry, leiomyoma, mesenchymal tumors, vaginal neoplasms

## Abstract

Angiolipoleiomyoma (ALLM) is a rare benign mesenchymal tumor consisting of mature adipose tissue, smooth muscle, and thick-walled blood vessels. Its occurrence in the female genital tract is uncommon, and vaginal involvement is exceptionally rare, making preoperative diagnosis particularly challenging due to its non-specific clinical and imaging features.

In this report, we present the case of a 45-year-old woman with a two-year history of progressive vaginal foreign body sensation accompanied by chronic constipation. Pelvic magnetic resonance imaging revealed a large mass measuring 13 × 9 × 8 cm in the rectouterine space, with superior displacement of the uterus. The patient underwent a total abdominal hysterectomy followed by a complementary vaginal resection of a tumor that was independent of the uterus. Histopathological examination revealed mature adipocytes, smooth muscle bundles, and thick-walled blood vessels. Immunohistochemical analysis showed positivity for desmin, smooth muscle actin, and CD34, and negativity for HMB-45. This confirmed the diagnosis of ALLM. The postoperative course was uneventful, with no recurrence observed at one-year follow-up. A literature review identified 47 publications comprising 68 reported cases of ALLM or extrarenal angiomyolipoma (AML) in the female genital tract, highlighting the extreme rarity of vaginal involvement.

This case underscores the diagnostic challenges associated with this entity and emphasizes the importance of histopathological and immunohistochemical evaluation in distinguishing it from other mesenchymal tumors.

## Introduction

Angiolipoleiomyoma (ALLM) is a rare benign mesenchymal tumor composed of varying proportions of mature adipose tissue, smooth muscle, and thick-walled blood vessels. Due to its histological similarity to renal angiomyolipoma (AML), some authors have referred to it as uterine AML [1]. However, despite these similarities, ALLM is considered a distinct entity, particularly in extrarenal locations, given its different immunohistochemical profile and clinical behavior.

This tumor is not formally included in the current World Health Organization (WHO) Classification of Female Genital Tumours (5th edition, 2020), which has led to inconsistent terminology in the literature, including designations such as hamartoma, lipoleiomyoma, and ALLM [2]. While lipoleiomyomas are generally regarded as variants of conventional leiomyomas with adipocytic metaplasia and AMLs are classified within the spectrum of perivascular epithelioid cell tumors (PEComas), ALLM remains a poorly defined entity that does not consistently fit into either category, particularly due to its typical lack of melanocytic marker expression, such as HMB-45. Its estimated incidence is extremely low, representing approximately 0.06% of benign uterine lesions [3], and it predominantly affects women over 40 years of age [4]. Unlike renal AMLs, uterine and extrarenal ALLMs are generally not associated with tuberous sclerosis and typically demonstrate negative immunoreactivity for HMB-45 [5].

Clinically, ALLM often presents with nonspecific symptoms such as pelvic pain, abnormal uterine bleeding, urinary complaints, or mass effect, depending on its size and anatomical location. These features frequently lead to misdiagnosis as more common gynecological conditions, including leiomyomas or complex adnexal masses [6]. Furthermore, imaging modalities such as ultrasound, computed tomography, and magnetic resonance imaging may reveal heterogeneous lesions with adipose components but lack pathognomonic features, making preoperative diagnosis particularly challenging. From a pathogenetic perspective, the origin of these tumors remains controversial, with hypotheses including adipocytic metaplasia within smooth muscle tumors, hamartomatous development, or a relationship with the PEComa family at the molecular level.

Although most reported cases arise in the uterine corpus, involvement of other structures within the female genital tract, including the cervix, broad ligament, ovary, and vulva, has been described. In contrast, vaginal localization is exceptionally rare, with only a limited number of cases reported in the literature, estimated to be fewer than 10 cases to date. In the literature review conducted as part of this study, 47 publications comprising 68 cases of ALLM or extrarenal AML in the female genital tract were identified. This rarity, combined with the absence of standardized diagnostic criteria, further complicates clinical recognition and management.

Given these considerations, we present a rare case of ALLM arising from the vaginal wall, emphasizing its clinical presentation, radiological findings, surgical management, and histopathological characteristics, along with a review of the literature to contextualize this uncommon entity.

## Case presentation

A 45-year-old woman, gravida 1, para 1, with a history of cesarean section, presented to the Gynecology Department with a progressive sensation of a vaginal foreign body associated with worsening chronic constipation of approximately two years’ duration with only partial response to medical treatment. She denied any other relevant personal or family medical history.

On gynecological examination, a mass arising from the posterior vaginal wall was identified. The lesion protruded beyond the vaginal introitus and hindered adequate speculum examination. On vaginal palpation, the cervix could not be identified. A rectal examination was therefore performed, revealing a mobile mass measuring approximately 10 cm in diameter and apparently occupying the pouch of Douglas.

**Figure 1:**
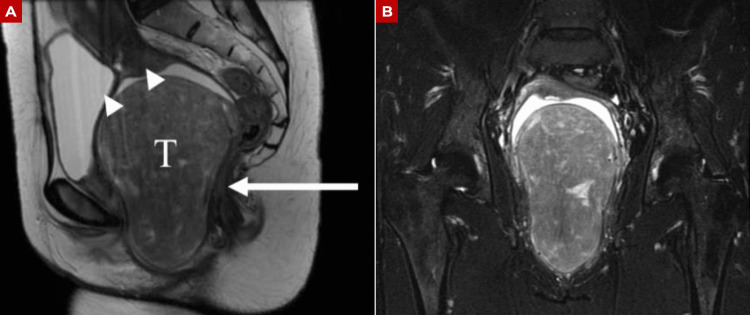
Pelvic magnetic resonance imaging demonstrating a large rectouterine mass with vaginal involvement (A) Sagittal T2-weighted image showing a large, heterogeneous mass occupying the rectouterine space, with loss of the normal interface with the posterior vaginal wall (arrowheads). The lesion (T) extends inferiorly toward the vaginal canal and displaces adjacent pelvic structures, including the uterus and bladder (arrow). (B) Coronal short tau inversion recovery (STIR) image demonstrating a well-defined, hyperintense mass occupying the central pelvis and extending toward the vaginal compartment, suggestive of a lesion with mixed soft tissue and adipose components.

As part of the diagnostic workup, pelvic magnetic resonance imaging revealed a space-occupying lesion measuring 13 × 9 × 8 cm in the rectouterine space, with superior displacement of the uterus (Figures 1A-1B). 

**Figure 2:**
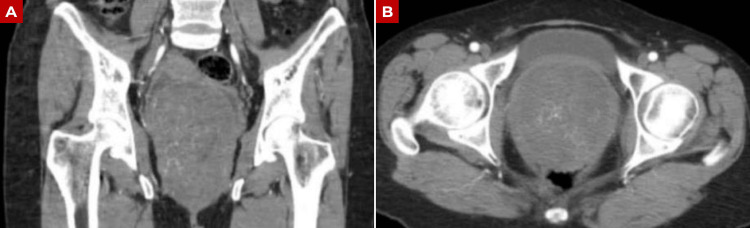
Contrast-enhanced abdominopelvic computed tomography demonstrating a large pelvic mass in relation to the posterior vaginal compartment (A) Coronal view showing a large, well-defined mass occupying the rectouterine space, with displacement of adjacent structures, including the uterus. (B) Axial view demonstrating a heterogeneous lesion with soft tissue attenuation located posterior to the bladder and closely related to the vaginal canal, compatible with origin from the posterior vaginal compartment.

These findings were further supported by plain and contrast-enhanced abdominopelvic computed tomography, which confirmed the presence of a large pelvic mass in close relationship with the posterior vaginal compartment (Figures 2A-2B). Based on the clinical and radiological findings suggestive of a pelvic neoplasm, a biopsy of the palpable lesion was performed. Histopathological examination of the biopsy specimen reported fragments compatible with leiomyoma. This discrepancy may be explained by sampling limitations inherent to biopsy procedures, particularly in heterogeneous tumors where limited tissue fragments may not be representative of the full histological composition of the lesion.

Given the progressive nature of the symptoms, the large size of the lesion, and the imaging findings suggestive of a pelvic neoplasm with uncertain origin, surgical management was indicated due to the need for definitive diagnosis and complete excision, as well as the potential for malignancy. The procedure was initially approached via laparotomy, and a total hysterectomy was performed, considering the close anatomical relationship of the mass with the uterus and the difficulty in excluding uterine involvement preoperatively. Intraoperatively, after removal of the uterine body and cervix, a distinct tumor was identified that was not dependent on either structure but originated from the posterior vaginal wall. Due to the difficulty in achieving complete excision through the abdominal approach alone, the procedure was complemented with a vaginal approach, allowing complete resection of the mass.

**Figure 3:**
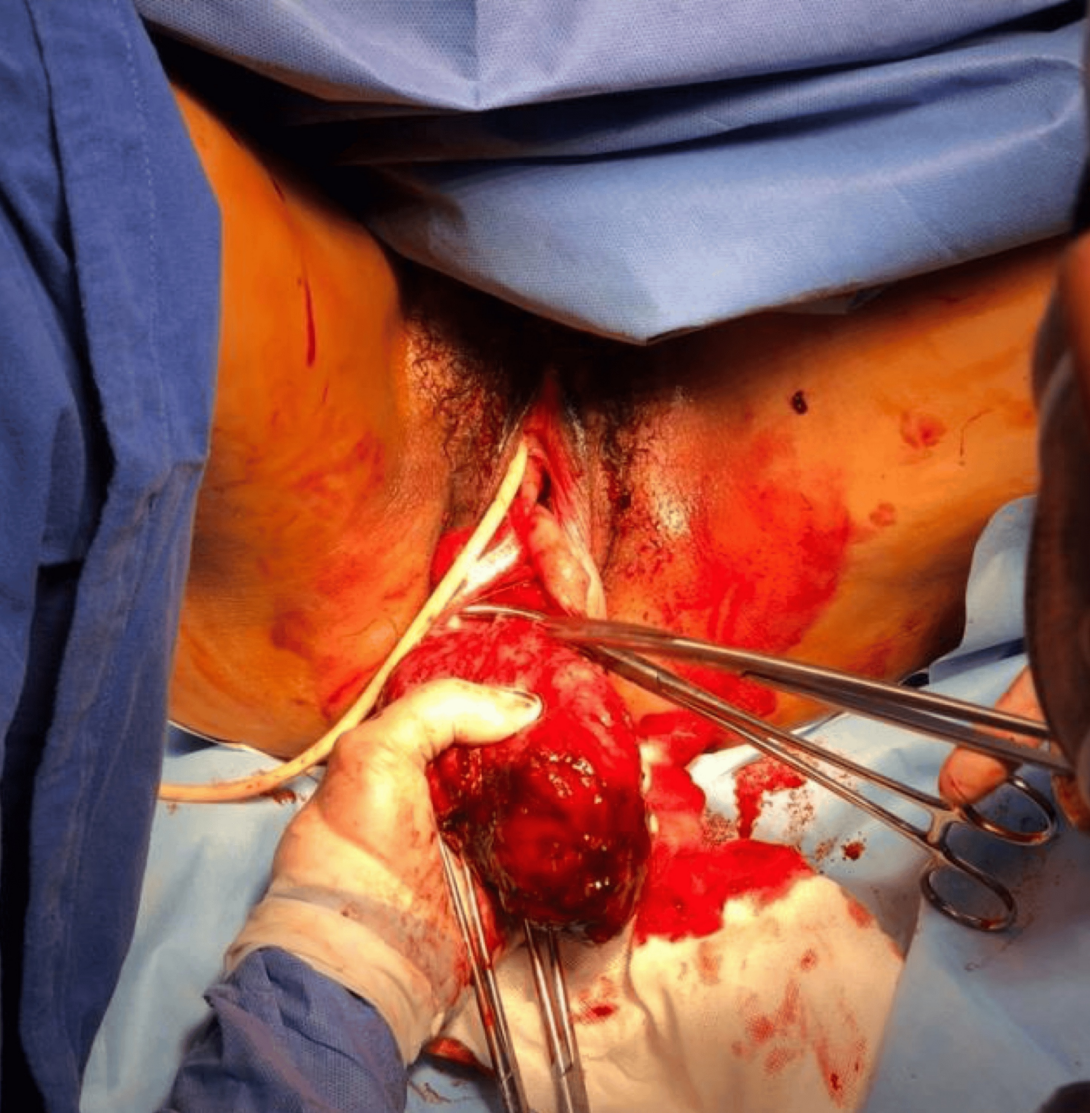
Intraoperative view of vaginal tumor resection Intraoperative photograph showing the surgical field during transvaginal excision of a well-circumscribed pelvic mass originating from the posterior vaginal wall. The lesion is observed being mobilized and dissected from surrounding tissues, demonstrating its encapsulated appearance and vascularized surface.

**Figure 4:**
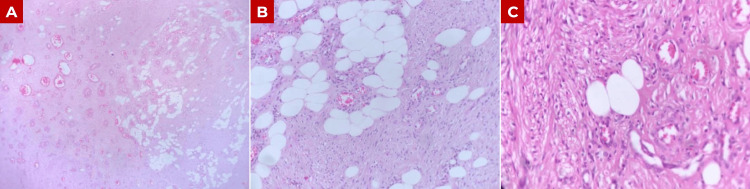
Histopathological features of angiolipoleiomyoma (A) Low-power magnification (40×) showing a well-circumscribed lesion composed of intermixed adipose tissue and smooth muscle. (B) Medium-power magnification (100×) revealing mature adipocytes interspersed among bundles of smooth muscle. (C) High-power magnification (400×) highlighting thick-walled blood vessels surrounded by smooth muscle cells, without cytological atypia or mitotic activity.

**Figure 5:**
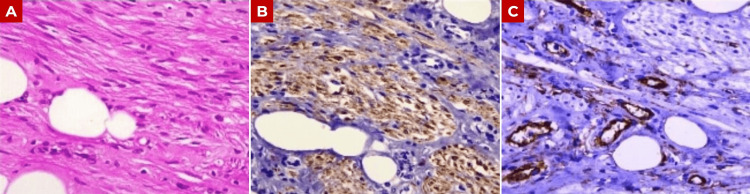
Immunohistochemical profile of angiolipoleiomyoma (A) Smooth muscle bundles with strong cytoplasmic positivity for smooth muscle actin (SMA), supporting smooth muscle differentiation. (B) Diffuse cytoplasmic positivity for desmin within the smooth muscle component. (C) CD34 positivity, highlighting the vascular endothelium, supporting the prominent vascular component of the lesion.

Gross examination revealed a firm, rubbery tumor measuring 12.2 × 7.5 × 4.5 cm and weighing 240 g (Figure 3). Histopathological evaluation demonstrated a lesion composed of mature adipose tissue, thick-walled blood vessels, and interlacing bundles of smooth muscle (Figure 4). No areas of necrosis, significant cytological atypia, or increased mitotic activity were identified. Immunohistochemical analysis showed positivity for desmin and smooth muscle actin in more than 75% of tumor cells, as well as CD34 positivity restricted to the vascular endothelium, supporting the prominent vascular component but not serving as a defining diagnostic marker. The tumor was negative for HMB-45, and although Melan-A and MiTF were not expressed, their absence further supports the exclusion of PEComa. These findings confirmed the diagnosis of ALLM (Figure 5 and Table 1).

**Table 1:** Immunohistochemical findings

Antibody	Clone	Reactivity	Intensity
Smooth muscle actin	1A4	Positive	>75% (++)
Desmin	EP15	Positive	>75% (++)
HMB-45	Hmb45	Negative	-
CD34	QBEND10	Positive	(++)

The postoperative course was uneventful. The patient attended routine follow-up visits and remained asymptomatic, with satisfactory clinical recovery and no evidence of recurrence at one year, confirming a favorable outcome following complete surgical resection.

## Discussion

Uterine ALLMs are rare benign mixed mesenchymal tumors composed of adipose tissue, smooth muscle, and vascular elements in variable proportions [2]. Over the last two decades, they have been associated with the spectrum of PEComas, although they are usually distinguished immunohistochemically by the absence of HMB-45 expression in most uterine and extrarenal cases [5].

Although extrarenal AMLs have been described in multiple organs, their occurrence in the female genital tract remains exceptional [7]. The first uterine case of this type of tumor was reported by McKeithen et al. in 1964 [8]. Different reviews have estimated that approximately 60-70 cases have been described in the literature [2].

To better contextualize the present case, a bibliographic search was performed in MEDLINE via PubMed, Scopus, The Lancet, EBSCO, and Google Scholar. The search was conducted up to January 2026 using the following terms: (“angiolipoleiomyoma of the vagina” OR “vaginal angiolipoleiomyoma” OR “vaginal angiomyolipoma” OR “angiomyolipoma of the uterus” OR “uterine angiolipoleiomyoma”). Review articles, case reports, and case series published in English and Spanish were included without restriction by year of publication. Animal studies and reports describing ALLMs outside the female genital tract were excluded. Duplicate records were removed, and studies were screened based on title and abstract, followed by full-text evaluation to confirm eligibility.

In the literature review conducted as part of this study, 47 publications documenting 68 cases of ALLM or extrarenal AML in the female genital tract were identified. The difference between the number of publications and the total number of cases is explained by the fact that some articles reported more than one case. These data are summarized in Table 2.

**Table 2:** Reported cases of angiolipoleiomyoma and extrarenal angiomyolipoma in the female genital tract References correspond to citation numbering in the text.

Author	Journal	Location	HMB-45
Seo et al. (2022) [1]	Curr Med Imaging	Uterine corpus	Negative
Psomiadou et al. (2022) [2]	Folia Med (Plovdiv)	Uterine corpus	Negative
Verocq et al. (2022) [3]	Int J Gynecol Pathol	Uterine corpus	Negative
Gupta et al. (2024) [4]	J Midlife Health	Uterine corpus/Cervix	-
Paryani and Shahid (2020) [5]	J Pak Med Assoc	Uterine corpus	Not performed
Poveda-Rojas et al. (2016) [6]	Rev Colomb Obstet Ginecol	Uterine corpus	Negative
Muniraj et al. (2021) [7]	Indian J Med Paediatr Oncol	Cervix	Negative
McKeithen et al. (1964) [8]	Obstet Gynecol	7 cases in uterine corpus	Not performed
Yahaya et al. (2025) [9]	Clin Case Rep	Ovary	Negative
Mulchandani and Vimala (2020) [10]	Indian J Gynecol Oncol	Uterine corpus	Negative
Marçal et al. (2022) [11]	Int J Med Sci Health Res	Vulva	Negative
Sieiński (1989) [12]	Int J Gynecol Pathol	Uterus/Cervix	Not performed
Kiran et al. (2023) [13]	Cureus	Cervix	Negative
Braun et al. (2002) [14]	J Clin Ultrasound	Uterine corpus	Not performed
Jacobs et al. (1965) [15]	Am J Clin Pathol	3 cases of uterine corpus	Not performed
Demopoulos et al. (1973) [16]	Am J Clin Pathol	4 cases of uterine corpus	Not performed
Burrows and Stroup (1975) [17]	Am J Obstet Gynecol	Uterine corpus	Not performed
Waxman et al. (1982) [18]	Am J Clin Pathol	Uterine corpus	Not performed
Katz et al. (1984) [19]	Am J Obstet Gynecol	Fallopian tube	Negative
Lo Re et al. (1987) [20]	Appl Pathol	Uterine corpus	Not performed
Peh and Sivanesaratnam (1988) [21]	Br J Obstet Gynaecol	Vaginal wall	Not performed
Chen (1990) [22]	Gynecol Oncol	Vaginal wall	Not performed
Laffargue et al. (1993) [23]	Gynecol Oncol	Uterine corpus	Negative
Shintaku (1996) [24]	Pathol Int	Uterine corpus	Not performed
Huang et al. (2000) [25]	Zhonghua Yi Xue Za Zhi	Cervix	Negative
Chetty and Pillay (2000) [26]	J Gynecol Surg	Uterine corpus	Not performed
Yaegashi et al. (2001) [27]	Pathol Int	Uterine corpus	Negative
Anderson et al. (2002) [28]	Int J Gynecol Pathol	Ovary	Positive
Ren and Wu (2004) [29]	Arch Pathol Lab Med	Uterine corpus	Negative
Cil et al. (2004) [30]	Gynecol Oncol	Uterine corpus	Positive (ET)
Daraï et al. (2004) [31]	J Reprod Med	Uterine corpus	Negative
Sarma et al. (2006) [32]	Internet J Pathol	Ovary	Positive
Cho et al. (2009) [33]	J Womens Med	Uterine corpus	Negative
An et al. (2010) [34]	J Womens Med	Parametrium	Negative
Kajo et al. (2010) [35]	Ceska Gynekol	Uterine corpus	Negative
Shakuntala et al. (2012) [36]	Indian J Surg Oncol	Broad ligament	Negative
Yilmaz et al. (2013) [37]	Electron J Gen Med	Uterine corpus	Negative
Lee et al. (2013) [38]	Eur J Gynaecol Oncol	Uterine corpus	Positive (ET)
Totev et al. (2014) [39]	J Biomed Clin Res	Uterine corpus	Negative
Garg et al. (2015) [40]	J Cancer Res Ther	Vulva	Not performed
Bacanakgil et al. (2022) [41]	Ginekol Pol	Uterine corpus	Negative
Shakarwal et al. (2017) [42]	Indian Obstet Gynaecol	Broad ligament	Not performed
Demir et al. (2018) [43]	Med J Bakirkoy	Uterine corpus	Not performed
Monteiro et al. (2019) [44]	Int J Res Med Sci	Cervix	Negative
Walke et al. (2019) [45]	J Med Sci Clin Res	Cervix	Not performed
Dutta et al. (2020) [46]	Autops Case Rep	Broad ligament	Negative
Wang et al. (2021) [47]	Arch Gynecol Obstet	Multiple sites	Mixed

Although Table 2 provides a descriptive summary of reported cases, several patterns can be identified. Most lesions arise in the uterine corpus, with fewer cases reported in the cervix, ovary, vulva, and broad ligament, and only exceptionally in the vaginal wall. The majority of patients are middle-aged women, typically over 40 years of age. Tumor size is variable, ranging from small incidental findings to large masses exceeding 10 cm, as observed in the present case. Histopathologically, all cases share a characteristic admixture of adipose tissue, smooth muscle, and vascular components, although immunohistochemical profiles may vary. Surgical excision remains the mainstay of treatment, with most reported cases demonstrating favorable outcomes and no evidence of recurrence during follow-up. Compared to previously reported cases, the present tumor is notable for its vaginal origin and large size, reinforcing the diagnostic challenges associated with this rare location.

The terminology used to describe these lesions remains controversial. Some authors have classified them as hamartomas, others as lipoleiomyomas, whereas others support the term uterine ALLM [10,15,16]. This lack of consensus is reinforced by the fact that the WHO does not explicitly recognize this lesion as an independent category within the classification of gynecological tumors, which limits diagnostic standardization and comparison across studies [11].

Macroscopically, ALLMs are usually well-circumscribed or encapsulated lesions with a firm or rubbery consistency and a pink-to-grey cut surface [3]. Tumor size in the literature is variable, with an average of approximately 8.4 cm, although lesions ranging from 2 to 16 cm have been reported [2,3]. Histologically, they show an admixture of mature adipose tissue, smooth muscle bundles, and thick-walled blood vessels, often with an irregular or tortuous configuration [3,24]. In the present case, histopathological examination demonstrated these characteristic findings, including mature adipocytes, interlacing smooth muscle bundles, and thick-walled vascular structures, without necrosis, cytological atypia, or significant mitotic activity (Figure 4).

When these tumors arise in the vaginal wall, they may present clinically with symptoms such as foreign body sensation, dyspareunia, pelvic pain, or abnormal genital bleeding, as described in previously reported vaginal cases [21,22]. Compared with those reports, our patient presented with a larger lesion and required a combined abdominal and vaginal surgical approach to achieve complete resection.

The differential diagnosis of ALLM includes lipoleiomyoma, vascular leiomyoma, PEComas, and, in some cases, teratoma. In this context, immunohistochemistry is essential for appropriate distinction, as ALLM is characterized by negative HMB-45 staining and positivity for muscle markers such as smooth muscle actin and desmin, as well as CD34, depending on the predominant tumor component [2,14,30]. In our case, immunohistochemical analysis showed positivity for desmin and smooth muscle actin in more than 75% of tumor cells, CD34 positivity in the vascular endothelium, and negativity for HMB-45 (Figure 5 and Table 1).

The distinction from PEComas, particularly AML, is especially relevant. These tumors are composed of perivascular epithelioid cells and typically show immunoreactivity for both muscle markers and melanocytic markers, especially HMB-45 [14,28]. By contrast, the absence of HMB-45 expression in ALLM constitutes a key criterion for differential diagnosis [2,5]. Nevertheless, HMB-45 positivity has been reported in a small number of cases, mainly in patients with tuberous sclerosis [30,32,41], suggesting possible histopathological overlap with true AMLs. At the molecular level, PEComas are frequently associated with alterations in the TSC1 and TSC2 genes, leading to activation of the mTOR signaling pathway, which plays a central role in tumorigenesis. In contrast, ALLMs typically lack these molecular alterations, supporting their distinction as a separate entity despite partial morphological overlap [30].

Because of these immunohistochemical differences, some authors have proposed that certain lesions may have been misclassified as PEComas when they actually corresponded to ALLM, which could contribute to the underestimation of their real prevalence [2,3]. Regarding histogenesis, Sieiński proposed several hypotheses, including embryonic mesodermal remnants, adipose metaplasia, or migration of pluripotent perivascular cells [12,13].

Imaging modalities such as ultrasound, computed tomography, and magnetic resonance imaging may identify heterogeneous masses with a fatty component, but they lack diagnostic specificity [1,14,15]. In our patient, imaging demonstrated a large rectouterine mass with superior displacement of the uterus and close relationship with the posterior vaginal wall (Figures 1-3), while preoperative biopsy suggested leiomyoma. This illustrates the difficulty of establishing an accurate diagnosis before surgery and supports the continued role of histopathological examination with immunohistochemistry as the diagnostic standard.

With regard to treatment, surgical resection remains the most frequently reported management strategy, particularly in symptomatic patients or in those with large lesions [2,4,11,30]. However, due to the rarity of ALLM, there is a lack of robust data regarding recurrence rates and long-term outcomes, and current evidence is largely derived from isolated case reports and small series.

In selected asymptomatic patients with small, well-circumscribed tumors, conservative management has been proposed; nevertheless, this approach is not well established and lacks sufficient longitudinal follow-up data to support its safety [2,11].

In the present case, complete excision required a combined abdominal and vaginal approach due to tumor size and anatomical location. The postoperative course was uneventful, and no recurrence has been observed during the one-year follow-up; however, longer surveillance is warranted to more definitively assess long-term outcomes.

## Conclusions

ALLM is an exceptionally rare benign mesenchymal tumor of the female genital tract, particularly when arising from the vaginal wall. Its diagnosis remains challenging due to non-specific clinical and imaging findings, which may lead to misinterpretation as more common pelvic masses such as leiomyomas or adnexal tumors.

This case highlights key diagnostic pitfalls, including the limitations of preoperative imaging and biopsy, as well as the potential for overlap with other adipose-containing mesenchymal tumors, particularly PEComas. In this context, a comprehensive histopathological and immunohistochemical evaluation is essential to establish a definitive diagnosis and guide appropriate management. Complete surgical excision remains both a diagnostic and therapeutic approach in symptomatic or large lesions. Importantly, this case underscores the value of a multidisciplinary approach integrating clinical, radiological, surgical, and pathological findings to improve diagnostic accuracy and optimize patient management in rare pelvic tumors.

## References

[1] Seo G, Park J, Lee E (2022). Uterine angiolipoleiomyoma with US, CT, and MRI findings: a case report. Curr Med Imaging.

[2] Psomiadou V, Iavazzo C, Geramani E (2022). Uterine angiolipoleiomyoma. A case report and systematic literature review. Folia Med (Plovdiv).

[3] Verocq C, Noël JC, Ouertani S, D'Haene N, Catteau X (2022). First case report of a uterine angiolipoleiomyoma with KRAS and KIT mutations. Int J Gynecol Pathol.

[4] Gupta D, Malhotra P, Rahar S, Ahuja A (2024). Angiolipoleiomyoma of the uterus with a dermoid cyst in the right ovary: an unusual association. J Midlife Health.

[5] Paryani NS, Shahid R (2020). Unsuspected components of a fibroid uterus: angiolipoleiomyoma. J Pak Med Assoc.

[6] Poveda-Rojas DC, Díaz-Gómez BL, Buriticá-Cifuentes C, García-Burgos AY, Alvarado-Heine C (2016). Uterine angiolipoleiomyoma: a case report and literature review (Article in Spanish). Rev Colomb Obstet Ginecol.

[7] Muniraj F, Divyapriya C, Raghavan V, Radha RK, Ramanujam S, Rajakumar SA (2021). Cervical angiomyolipoma coexisting with endometrial carcinoma in the absence of tuberous sclerosis: a rare case report. Indian J Med Paediatr Oncol.

[8] McKeithen W, Shinner J, Michelsen J (1964). Hamartoma of the uterus. Obstet Gynecol.

[9] Yahaya JJ, Morgan ED, Nyakato V, Othieno E (2025). Incidental primary angiomyolipoma of ovary: a rare case report and literature review. Clin Case Rep.

[10] Mulchandani NJ, Vimala R (2020). Uterine angiomyolipoma: a case report and review of literature. Indian J Gynecol Oncolog.

[11] Marçal JMB, Vieira LFC, Wilke EDA (2022). Intrinsic features of vulvar angioleiomyoma: case report and mini review. Int J Med Sci Health Res.

[12] Sieiński W (1989). Lipomatous neometaplasia of the uterus. Report of 11 cases with discussion of histogenesis and pathogenesis. Int J Gynecol Pathol.

[13] Kiran N, Ramanarasimhaiah R, Khan S, Mody K (2023). Angiomyolipoma of uterine cervix: report of a rare case. Cureus.

[14] Braun HL, Wheelock JB, Amaker BH, Seeds JW (2002). Sonographic evaluation of a uterine angiolipoleiomyoma. J Clin Ultrasound.

[15] Jacobs DS, Cohen H, Johnson JS (1965). Lıpoleıomyomas of the uterus. Am J Clin Pathol.

[16] Demopoulos RI, Denarvaez F, Kaji V (1973). Benign mixed mesodermal tumors of the uterus: a histogenetic study. Am J Clin Pathol.

[17] Burrows S, Stroup PE (1975). Benign mixed mesodermal tumor in the uterus presenting at delivery. Am J Obstet Gynecol.

[18] Waxman M, Boyce JG, Macasaet MM, Lu T (1982). Concurrence of malignant and benign heterologous mixed tumors of the uterus. Am J Clin Pathol.

[19] Katz DA, Thom D, Bogard P, Dermer MS (1984). Angiomyolipoma of the fallopian tube. Am J Obstet Gynecol.

[20] Lo Re V, Santangelo M, Fibbi ML, Spinelli M, Canevini P (1987). Benign lipomatous lesions of the uterus: 3 new cases, review of the literature and histogenetic considerations. Appl Pathol.

[21] Peh SC, Sivanesaratnam V (1988). Angiomyolipoma of the vagina--an uncommon tumour. Case report. Br J Obstet Gynaecol.

[22] Chen KT (1990). Angiomyolipoma of the vagina. Gynecol Oncol.

[23] Laffargue F, Giacalone PL, Charpin C, Lachard A (1993). Uterine angiomyolipoma associated with pregnancy. Gynecol Oncol.

[24] Shintaku M (1996). Lipoleiomyomatous tumors of the uterus: a heterogeneous group? Histopathological study of five cases. Pathol Int.

[25] Huang PC, Chen JT, Ho WL (2000). Clinicopathologic analysis of renal and extrarenal angiomyolipomas: report of 44 cases. Zhonghua Yi Xue Za Zhi (Taipei).

[26] Chetty R, Pillay P (2000). Sporadic angiomyolipoma of the uterus. J Gynecol Surg.

[27] Yaegashi H, Moriya T, Soeda S, Yonemoto Y, Nagura H, Sasano H (2001). Uterine angiomyolipoma: case report and review of the literature. Pathol Int.

[28] Anderson AE, Yang X, Young RH (2002). Epithelioid angiomyolipoma of the ovary: a case report and literature review. Int J Gynecol Pathol.

[29] Ren RL, Wu HH (2004). Pathologic quiz case: a 40-year-old woman with an unusual uterine tumor. Uterine angiolipoleiomyoma with focal atypical leiomyoma. Arch Pathol Lab Med.

[30] Cil AP, Haberal A, Hucumenoglu S, Kovalak EE, Gunes M (2004). Angiomyolipoma of the uterus associated with tuberous sclerosis: case report and review of the literature. Gynecol Oncol.

[31] Daraï E, Bazot M, Barranger E, Detchev R, Cortez A (2004). Epithelioid angiomyolipoma of the uterus: a case report. J Reprod Med.

[32] Sarma N, Jeebun N, Gopalakrishnan V (2006). Primary angiomyolipoma of the ovary. Internet J Pathol.

[33] Cho H, Kim H, Lee M, Kim K (2009). Uterine angiomyolipoma with partial cystic changes. J Womens Med.

[34] An J, Kim HN, Cho HY, Chung DH, Kim NR (2010). Uterine vascular leiomyoma with fat component. J Womens Med.

[35] Kajo K, Zúbor P, Krivus S, Danko J (2010). Angiolipoleiomyoma of the uterus. Case report and literature (Article in Slovak). Ceska Gynekol.

[36] Shakuntala PN, Shilpashree M, Geethanjali S, Sharma SK (2012). Acute abdomen as an unusual presentation of broad ligament angiomyolipoma. A case report and review of literature. Indian J Surg Oncol.

[37] Yilmaz M, Ingec M, Isaoğlu U, Sipal S (2013). Angiomyolipoma of the uterus. Electron J Gen Med.

[38] Lee SJ, Yoo JY, Yoo SH, Seo YH, Yoon JH (2013). Uterine angiomyolipoma with metastasis. Eur J Gynaecol Oncol.

[39] Totev TP, Mateva SA, Nikolova MR, Gorchev GA (2014). Uterine angiomyolipoma. J Biomed Clin Res.

[40] Garg M, Duhan A, Bindroo S, Kaur J, Mahajan NC (2015). Isolated angiomyolipoma of vulva: a case report of an uncommon tumor at an uncommon site. J Cancer Res Ther.

[41] Bacanakgil BH, Ilhan G, Kaban I (2022). Variant type of leiomyomas: 13 years of experience in a single institution. Ginekol Pol.

[42] Shakarwal DS, Agrawal DS, Chopra DK, Raghunandan DC (2017). Pseudo-broad ligament angiolipoleiomyoma mimicking ovarian torsion - a rare case report. Indian Obstet Gynaecol.

[43] Demir Cendek B, Avsar AF, Bostanci Ergen E, Orhun Yavuz HS, Bedir Findik R (2018). Rarely seen benign tumor of the uterus, angiolipoleiomyoma: a case report. Med J Bakirkoy.

[44] Monteiro R, Sharma S, Gupta S, Choudhary I (2019). Extrarenal angiomyolipoma in uterine cervix: rare presentation in unusual site. Int J Res Med Sci.

[45] Walke V, Gaikwad A, Ratnaparkhi S, Kowe B (2019). Angiomyolipoma of the cervix. J Med Sci Clin Res.

[46] Dutta S, Marbaniang E, Dey B, Lyngdoh BS, Raphael V (2020). Angiomyolipoma of the broad ligament. Autops Case Rep.

[47] Wang J, Yang Q, Zhang N, Wang D (2021). Uterine angiomyolipoma: a clinical analysis of 8 cases and literature review. Arch Gynecol Obstet.

